# Circulating DNA tumor fraction as a biomarker for advanced breast cancer

**DOI:** 10.3389/fonc.2025.1655415

**Published:** 2025-11-10

**Authors:** Emily Cybulla, Daniel G. Stover

**Affiliations:** 1 Ohio State University College of Medicine, Columbus, OH, United States; 2 The Ohio State University Comprehensive Cancer Center, Arthur G. James Cancer Hospital and Richard J. Solove Research Institute, Columbus, OH, United States; 3 Stefanie Spielman Comprehensive Breast Center, Columbus, OH, United States

**Keywords:** liquid biopsy, breast cancer, metastatic cancer, biomarker, cancer

## Abstract

Liquid biopsy has emerged as an important clinical tool for managing a range of tumor types, including advanced breast cancers. Early work in metastatic breast cancer (MBC) demonstrated that circulating tumor DNA (ctDNA) could be reliably detected in patient peripheral blood samples. These discoveries laid the groundwork for more recent work monitoring the circulating DNA tumor fraction, which is the fraction of ctDNA among total cell-free DNA. Studies have shown tumor fraction to be a useful prognostic biomarker in advanced breast cancer. Clinical trials have also begun to explore tumor fraction’s role in predicting response to specific therapeutic agents in MBC. Future investigation will be critical to understand when and how tumor fraction should inform clinical decision-making for breast cancer patients in a variety of settings, especially given the increasing accessibility of both commercial and research-based assays that report tumor fraction.

## Introduction

Incidence of metastatic breast cancer (MBC) in the United States has increased in the last two decades (1), and an estimated 20-30% of patients diagnosed with early-stage breast cancer will develop metastatic disease (2). While the historical 5-year survival rate for patients with metastatic breast cancer in the United States has been previously estimated to be between 20-30% (3), the growing number of effective and tolerable therapies for MBC are leading patients to live longer with advanced disease. As such, developing prognostic and predictive biomarkers to support clinical decision-making for patients with advanced breast cancer is critical to improving these outcomes.

**Figure 1:**
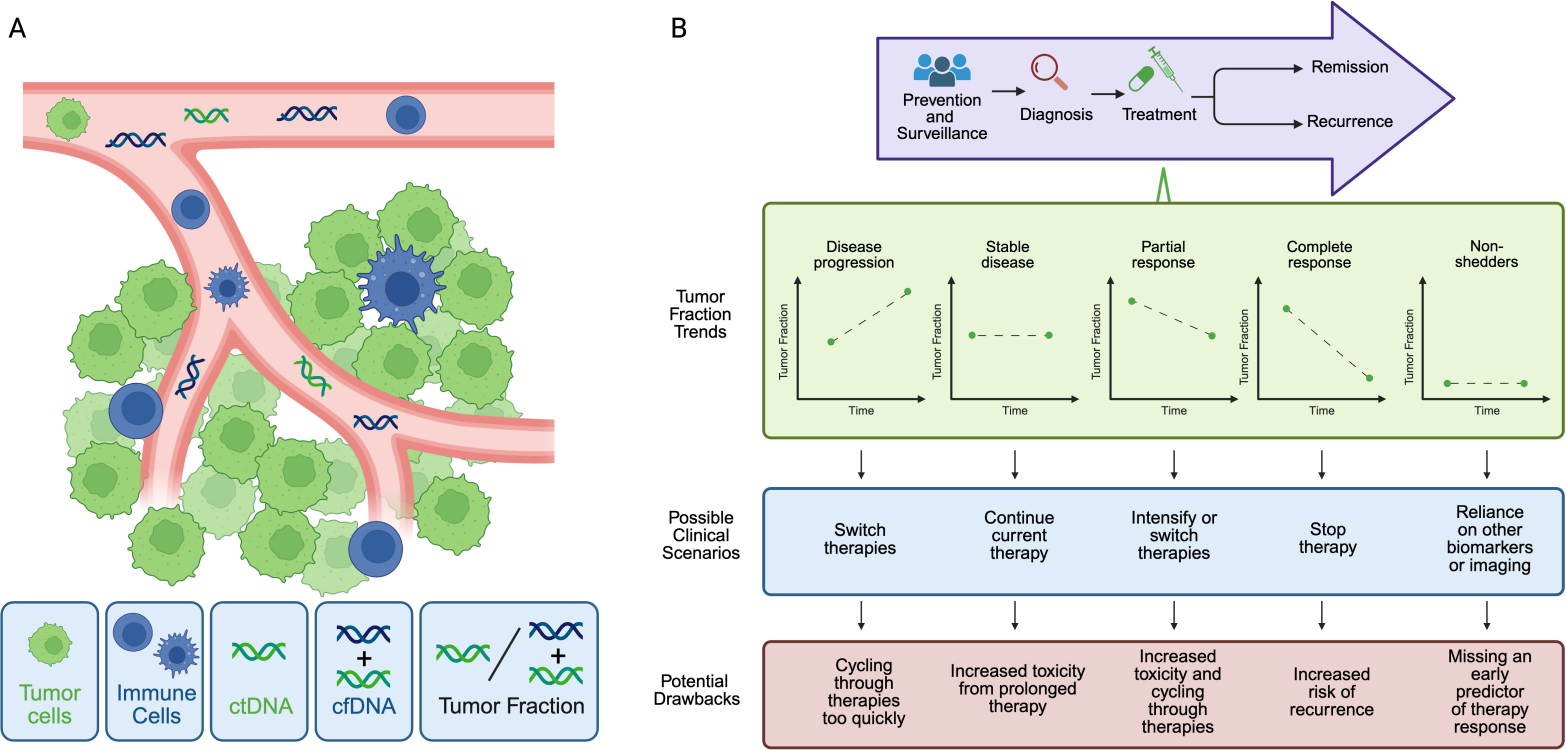
Tumor fraction in liquid biopsy. **(A)** Tumor cells (green) develop into solid malignancy with a microenvironment composed of infiltrating immune cells (blue), vasculature (pink), endothelial cells and extracellular matrix (not shown). Circulating tumor cells can be detected in blood, as well as circulating tumor DNA (ctDNA, green DNA strand) and total cell free DNA (cfDNA, green plus blue DNA strands). cfDNA includes DNA that is released from non-cancer cells, such as immune cells. Tumor fraction is defined at the proportion of ctDNA relative to the total cfDNA. **(B)** Currently, tumor fraction is primarily used after diagnosis of advanced disease (top panel). During treatment, tumor fraction could correlate with response to diverse therapies and support clinical decision-making to escalate, switch, or continue a drug regimen. Before broad integration it is unclear whether tumor fraction change, in addition to tumor fraction at a given time point, is a useful predictive or prognostic biomarker in advanced breast cancer, particularly in with tumors that shed minimal ctDNA (low- or ‘non-shedders’), among others.

One strategy that has emerged to understand advanced breast tumor heterogeneity, evolution, and biology over time is monitoring of tumor fraction, which is the fraction of circulating tumor-derived DNA (ctDNA) among total cell-free DNA (cfDNA) (**Figure 1A**). Although important work has also explored detection of circulating tumor cells (CTCs), for the purposes of this review, we will focus our attention on tumor fraction. Several assay approaches have been developed to quantify tumor fraction in patient samples (**Table 1**). In ultra-low pass whole-genome sequencing, small fragment DNA is extracted from plasma, and whole genome sequencing is then performed at shallow coverage before computation of a tumor fraction. The limit of detection of ULP-WGS is ~1-3% (depending on depth of sequencing), meaning that detecting the presence of tumor in patient samples requires that at least 1% of the tumor fraction be present, which typically limits the utility of ULP-WGS to advanced/metastatic settings (4, 5). Relative to whole exome sequencing that requires around 50-100% of cfDNA to be used in the assay, ULP-WGS uses only a fraction of each plasma sample, offering the potential for other assays (e.g. targeted panel sequencing, whole exome or genome sequencing, methylation) on the remaining majority of the sample. Further, the low cost of sequencing for ULP-WGS means that in most cases a sample costs less than $100 to assay. There are also other approaches to evaluate tumor fraction. The variant allele frequency (VAF) or allele fraction (AF) approach involves determining the fraction of reads carrying a specific mutation, which serves as a surrogate marker for tumor fraction (6, 7). More sensitive approaches to detect ctDNA at levels orders of magnitude lower than ULP-WGS include deep WGS or personalized assays have been primarily applied in the early stage breast cancer setting, yet may have utility in advanced cancer settings with low circulating tumor DNA levels (**Table 1**).

**Table 1:** Tumor fraction assay comparison.

Technology	% Genome sequenced	Tumor content	Analysis	Cost	Turnaround time	Selected references
ULP-WGS	100%	TFx to ≈ 3%	Easy	$	Weeks	(4, 17, 22)
Genotyping/ddPCR	<0.001%	VAF	Easy	$	Days	(21, 25–27)
Targeted Panel	<0.1%	VAF	Moderate	$$	Weeks	(18–21, 28)
WES	1%	TFx to ≈ 0.1%	Moderate	$$	Weeks to months	(4)
WGS	100%	TFx to ≈ 1%	Complex	$$$	Months	(7)
Personal Assay	Pre: 1%-100%	TFx to <<0.1%	Complex	$$$	Variable	(23, 24)

Assays employed to determine tumor fraction vary in the percent of genome sequenced, tumor content, estimated turnaround time, analysis difficulty, and cost. Selected references utilizing each approach are also included in the final column. ULP-WGS, ultra-low pass whole genome sequencing; ddPCR, droplet digital polymerase chain reaction; WES, whole exome sequencing; WGS, whole genome sequencing.

Another consideration when evaluating utility of these assays is whether they are tumor-informed versus tumor-agnostic, as tumor-informed approaches require prior knowledge of patient’s specific tumor mutations that can be tracked over time, while tumor-agnostic assays rely on pre-determined panels of mutations (8). While tumor-informed assays tend to be more specific and sensitive, these methods are also more time-consuming, more cost-prohibitive, and require tissue biopsy to identify mutations of interest in patients (8). Data has shown that ULP-WGS provides accurate tumor fraction measurement when compared to WES (4, 5), and tumor fraction assessment by targeted panel correlates with tumor fractions measured by ULP-WGS (9). Cost, sensitivity, accuracy, turnaround time, and complexity of data analysis are all factors to consider when evaluating clinical applicability of these approaches in advanced breast cancer (**Table 1**).

There are also several commercial proprietary assays that have begun to report tumor fraction, along with other metrics including tumor mutational burden and microsatellite instability. Each assay evaluates a variable number of relevant genes with specificities and sensitivities reported for detection of single nucleotide variations, copy number alterations, fusions/rearrangements, and indels, depending on the particular assay (non-exhaustive list provided in **Supplementary Table 1**). Available published data highlights variable concordance or positive percent agreement (PPA) between these commercial liquid-based biopsies and matched tissue biopsies (10–13) (**Supplementary Table 1**).

## Historical perspective

Early studies on ctDNA were conducted across a variety of tumor subtypes and addressed several important questions related to the sensitivity of this approach, including whether tumor DNA could be detected in peripheral blood samples and whether the methods available could be used to evaluate tumor DNA metrics such as copy number alterations, somatic mutations, and mutational signatures. Heitzer et al. demonstrated that tumor-specific copy number alterations could be detected in peripheral blood samples of both colorectal and breast cancer patients (14). There was a biphasic distribution of plasma DNA size in samples from cancer patients, which was associated with circulating tumor cell occurrence (14). Authors also uncovered copy number differences between patient’s primary tumors and metastatic sites in their CRC samples, suggesting that plasma DNA could prove a useful approach to understanding tumor evolution and heterogeneity, both of which play integral roles in cancer metastasis (14). Similar work showed that concentrations of cell-free plasma DNA in breast cancer samples are elevated in patients with tumors>2cm relative to tumors<2cm and in patients with distant breast cancer metastasis when compared to those with nodal negative and nodal positive disease (15).

Another early study uncovered that ctDNA could be detected in metastatic breast cancer blood samples, alongside a serum biomarker CA 15-3 (16). Interestingly, ctDNA levels correlated with tumor burden more closely than CA 15–3 levels or circulating tumor cells (16). Adalsteinsson et al. also showed that ctDNA collected from peripheral blood samples could reliably be used for whole exome sequencing (4). In these breast cancer blood samples, mutational signatures, such as the APOBEC-related and homologous recombination (HR) deficiency-related signatures were also detected, as well as somatic mutations and copy number alterations (4). Importantly, these data illustrated high concordance between ctDNA and metastatic tumor whole-exome sequencing, which is crucial for operationalizing ctDNA into routine breast cancer clinical practice where tissue biopsy remains the gold-standard for evaluating disease (4). These studies laid the foundation for contemporary work exploring tumor fraction as a biomarker in advanced breast cancer.

## Tumor fraction as a prognostic biomarker

More recent work has elucidated that tumor fraction has prognostic significance in advanced breast cancer. In a retrospective cohort study of metastatic triple negative breast cancer patients, samples with tumor fraction >10% had a significantly lower survival probability relative to samples with tumor fraction <10% (17). Similarly, within a cohort of metastatic breast cancer patients with a variety of histologic subtypes, another study demonstrated that patients with tumor fraction >10% again had worse survival outcomes (18). This work also showed that TF remained prognostic in mBC with cutoff points ranging from 1% to 20% (18). Bader et al. also demonstrated that patients with metastatic breast cancer and low tumor fraction (<1%) had significantly improved real-world overall survival, compared to those with either intermediate tumor fraction (1-10%) or high tumor fraction (>10%) (19). Tumor fraction remained prognostic when analyzing patients with bone-only metastases in this advanced breast cancer population (19).

Another clinically relevant aspect of liquid biopsy is whether these assays can detect actionable mutations that would open the possibility of targeted therapies. In breast cancer, work has shown that a range of mutations, including PIK3CA, BRCA1/2, and PTEN, can be detected in liquid biopsy (20, 21). In one study, in patients with tumor fraction greater to or equal to 10%, the sensitivity of detecting BRCA1/2 mutations was reported as 86% (20). In addition to individual mutations detected in circulating tumor DNA, DNA-based signatures in metastatic breast cancer have also been developed based on liquid biopsy and are associated with different survival outcomes. One ctDNA-based multi-gene signature tracking RB-LOH gene expression signature was associated with different progression-free survival and overall survival probabilities (22). Metastatic breast cancer samples with high RB-LOH signature score had significantly decreased progression free and overall survival probability when compared to the medium or low RB-LOH signature cohorts, independent of tumor fraction (22). While tumor fraction – and potentially other ctDNA features - appear to be prognostic in diverse advanced cancer settings, rarely are clinical decisions made based on prognostic factors. Further, there are many prognostic factors for advanced breast cancer that range from patient-related features (e.g. ECOG Performance Status) to protein tumor markers (such as CA15–3 and CA19-9).

## Tumor fraction change as a predictive biomarker?

Additional studies have begun to establish that tumor fraction can be utilized to predict response to specific therapies beyond its value as a prognostic biomarker. There are a growing number of Phase II/III clinical trials incorporating or utilizing circulating tumor DNA (**Supplementary Figure 1**). One example of prior work is translational research from PALOMA-3, a phase III multicenter, double-blind randomized control trial of Palbociclib plus Fulvestrant vs placebo plus Fulvestrant in women with HR^+^/HER2^−^ advanced breast cancer. Although overall survival was improved in the Palbociclib plus Fulvestrant arm of PALOMA-3, patients with high tumor fraction (>10%) had worse progression-free survival in both treatment arms (Palbociclib plus Fulvestrant and placebo plus Fulvestrant) (23, 24). Further stratification of patients into high tumor fraction and low tumor fraction cohorts might inform whether patients would benefit from more aggressive therapy approaches or prolonged courses of treatment. Patients with specific mutations detected in ctDNA in PALOMA-3 also had different survival outcomes. In both treatment arms, patients with TP53 mutation or with FGFR1 gain had decreased progression free survival relative to TP53 WT and no FGFR1 gain patients who were receiving the same treatment (24). While these data provide early evidence that high vs. low tumor fraction might be associated with different survival outcomes in advanced breast cancer, the question remains of when, either before or during or after initiation of a therapy, tumor fraction might be the most accurate predictor of survival.

Another clinical trial, the BEECH study, aimed to answer this question by monitoring ctDNA levels over time in patients with ER+ metastatic breast cancer receiving paclitaxel plus placebo vs. paclitaxel plus an AKT inhibitor (capivasertib) (25). In follow-up analyses, authors defined a circulating tumor DNA ratio (CDR) as the ratio between on-treatment mutation fraction and baseline/screening mutation fraction and showed that C2D1 after initiation of therapy was the ideal timepoint to assess CDR and discriminate between long and short progression-free survival (26). Consistent with the PALOMA-3, trial, BEECH also showed that patients with high ctDNA (by measurement of CDR>0.25) had decreased PFS in both treatment and placebo arms. But broad application of the same timepoint identified by Hrebien et al. is limited by the likely impact of drug pharmacokinetics and underlying patient factors on ctDNA formation and clearance, which will need to be studied further across advanced breast tumor types, diverse patient cohorts, and different treatment paradigms.

Data from Bidard et al. in the PADA-1 clinical trial also provides some early insight into whether liquid biopsy can identify therapy resistance in ER+/HER- advanced breast cancers (27). Patients in the study were placed on a first-line aromatase inhibitor (AI) plus palbociclib and followed for increasing levels of ESR1 mutation in blood samples (bESR1_mut_), as ESR1 mutation has been associated with acquired resistance to AIs. Criteria for rising bESR1_mut_ were met when samples were negative for bESR1_mut_ before cycle #1 of therapy and became positive for bESR1_mut_ before cycle #2 or at subsequent cycles or when samples were positive for bESR1_mut_ before cycle #1 and remained positive at subsequent cycles with a mutant allele frequency (MAF) greater than or equal to 3 times the lowest MAF ever detected (27). Once rising bESR1_mut_ was observed, patients were randomized into continuation of therapy with AI plus palbociclib vs. switch to fulvestrant plus palbociclib (27). PFS was significantly longer in the fulvestrant plus palbociclib group relative to the group continued on previous therapy (27), suggesting that early transition to an alternative drug after detection of a resistance marker could confer survival benefit. Although not equivalent to tumor fraction, MAF could be considered a tumor fraction surrogate that has predictive value in metastatic breast cancer. In SERENA-6, authors also demonstrated that patients switched to camizestrant therapy had longer PFS relative to patients who remained on AI therapy after detection of ESR1 mutations before clinical or radiologic evidence of disease progression (28). These compelling studies do support liquid biopsy’s role in identifying early therapy resistance in breast cancer. However, one of the limitations of using MAF or single mutation detection as early biomarkers is that these approaches do not fully capture tumor heterogeneity so might overlook the multifactorial mechanisms that promote therapy resistance across breast tumor subtypes.

## Discussion

Liquid biopsy is emerging as a promising tool across the cancer care continuum. Within breast cancer, one could envision using liquid biopsy to aid in early detection of malignancy in the general population before imaging evidence of cancer appears. At time of diagnosis, liquid biopsy could then provide a minimally invasive approach to characterize the primary tumor and serially monitor dynamic tumor changes before treatment begins without the need for repeat tissue biopsies. During treatment, tumor fraction could correlate with response to diverse therapies and support clinical decision-making to escalate, switch, or continue a drug regimen (**Figure 1B**). Following breast cancer treatment, tumor fraction detection could also be used for minimal residual disease quantification and monitoring for potential breast cancer recurrence.

However, before broad integration of liquid biopsy into routine breast cancer patient care, several other outstanding questions remain in the field. First, it is unclear whether tumor fraction change, in addition to tumor fraction at a given time point, is a useful predictive or prognostic biomarker in advanced breast cancer. Second, does tumor fraction have utility in non-shedders or low-shedders (**Figure 1B**)? Non-shedding refers to no detectable ctDNA (tumor fraction = 0), based on the specific assay. Shedding levels and kinetics in solid tumors could be influenced by tumor-intrinsic and microenvironment factors, overall cancer burden, treatment type, and patient factors. Highly proliferative or large tumors and those that are surrounded by highly vascularized microenvironments might be more likely to shed high levels of ctDNA. ctDNA clearance by immune cells is also impacted by host variables, including age and other medical co-morbidities. In breast cancer, while there is evidence that the presence of liver metastasis is linked to increased ctDNA levels (17, 29), non-shedders have been identified in bone-only and non-bone only metastatic breast cancer cohorts. Frequency of non-shedding is also higher among ER+/HER2- breast cancers, relative to TNBC or HER2+ tumors (30). However, the exact mechanisms underpinning why certain tumor subtypes are non-shedding remains unknown. As a result, further investigation in non-shedders is necessary to determine the utility of tumor fraction in this patient population.

Another limitation of the current tumor fraction literature in advanced breast cancer is that most studies have been done in retrospective cohorts with small sample sizes, which does reduce statistical power and broader clinical applicability. Additionally, tumor fraction cutoff variability and differences in the extent of survival correlation make designation of a universal tumor fraction cutoff value challenging. Many studies discussed in this review have reported prognostic significance for a tumor fraction greater than 10%. Data collected by Adalsteinsson et al. also show that a tumor fraction of at least 10% is appropriate for standard coverage whole exome sequencing, which was utilized experimentally to assess concordance between liquid biopsy and tissue-based biopsy (4) and is a relevant clinical consideration. Some work has also shown that targeted panels in metastatic breast cancer samples with tumor fractions < 1% less reliably detect genomic alterations (31) and have lower positive percent agreement with tissue-based biopsy (32), raising possible limitations of lower tumor fraction cutoffs with certain assays. However, it remains to be established whether a tumor fraction of 10% or alternative thresholds will exhibit more consistent prognostic or predictive value in advanced breast cancer patients. Important future studies will also expand on previous work evaluating tumor fraction relative to other blood-based biomarkers (16, 33) that are independently associated with survival, including circulating tumor cells (CTCs) and protein-based biomarkers (e.g. CA15-3, CA27-29). Specifically, it will be important to determine of tumor fraction adds additional information to these alternative approaches.

It is also not yet clear whether the type of assay used to quantify tumor fraction affects the data trends or interpretation of these data. Importantly, the field needs to establish whether tumor fraction can or should be used to make therapeutic decisions for patients. Should physicians be changing therapies immediately based on tumor fraction, potentially before imaging evidence of resistance or recurrence? Or would this approach simply lead to cycling of therapies without enough time to assess for adequate response? And does the benefit of utilizing tumor fraction apply to all breast tumor types with a range of metastatic sites and across patient demographics? Ongoing integration of tumor fraction into clinical trial design is underway, with several interventional trials in metastatic breast cancer incorporating circulating tumor DNA approaches which may help shed light on these important outstanding questions in the field (**Supplementary Figure 1**).

Further investigation is also needed to assess the feasibility of using tumor fraction across diverse practice settings, including in under-resourced health systems in low and middle-income countries, and across diverse patient populations. Some exciting collaborative efforts in Ghana showed that ctDNA could be detected in a cohort of breast cancer patients across three hospitals in the country (34). Work has also shown that plasma DNA can be analyzed from dried blood spots, which would provide a convenient and economical approach to evaluate ctDNA and tumor fraction in settings with limited healthcare and research resources (35, 36). One retrospective study also showed that ctDNA was detectable across tumor types and tumor stages, including metastatic breast cancer, in a Southeast Asian patient cohort in Vietnam (37). Another recent review from Aronson et al. discusses how genetic ancestry impacts ctDNA detection rates and ctDNA genomic profiles, and the authors also detail barriers to equity in utilization of ctDNA testing across racially and ethnically underrepresented patient populations (38). The performance of tumor fraction as biomarker will rely on crucial research exploring ctDNA biology and tumor fraction testing implementation in these populations, especially when considering the clinical outcomes of advanced breast cancer. Within the United States, one retrospective study showed black patients and patients in the lowest socioeconomic status quintile had the highest mortality related to their metastatic breast cancer. Interestingly, in black patients with HR- metastatic breast cancer, living in rural areas was also associated with an increased risk of breast cancer mortality (39). Given that the burden of metastatic breast cancer disproportionately affects patients in low and middle-income countries, patients of color, and persons in lower socioeconomic quintiles and rural communities, liquid biopsy accessibility remains paramount to addressing cancer disparities and improving outcomes for all patients.

In conclusion, circulating DNA tumor fraction is increasingly available to clinicians as part of commercial tests and to researchers through a variety of assay types. While data regarding the prognostic significance in breast cancer is robust, the utility as a predictive biomarker – a critical clinical need – remains less well-defined. Further work on understanding patients with little or no detectable ctDNA and how to best implement tumor fraction-based strategies are needed to optimally incorporate tumor fraction into clinical practice for breast cancer care.

## Supplementary material

Supplementary Figure 1 Phase II/III Clinical trials Utilizing ctDNA. Forty-four Phase II or III interventional trials in metastatic breast cancer were identified. Of these trials, 32 were measuring ctDNA as one of their outcomes or monitoring ctDNA over time, and 12 trials proposed to utilize ctDNA measures up-front to inform the interventional component of the study. Trials with any phase 1 component, as well as terminated or withdrawn trials were not included.

Supplementary Table 1 Commercial liquid biopsy assay comparison. Examples of commercial assays vary in the number of genes evaluated and other reported metrics (not exhaustive list). The assays listed all report Tumor Fraction. Each assay has variable published concordance and positive percent agreement (PPA) data.
