# Supplementary figures and images for: Circulating DNA tumor fraction as a biomarker for advanced breast cancer
Source: Front Oncol. 2025 Nov 10;15:1655415. doi: 10.3389/fonc.2025.1655415 (PMC12640844; doi:10.3389/fonc.2025.1655415)

Supplementary Figure 1.

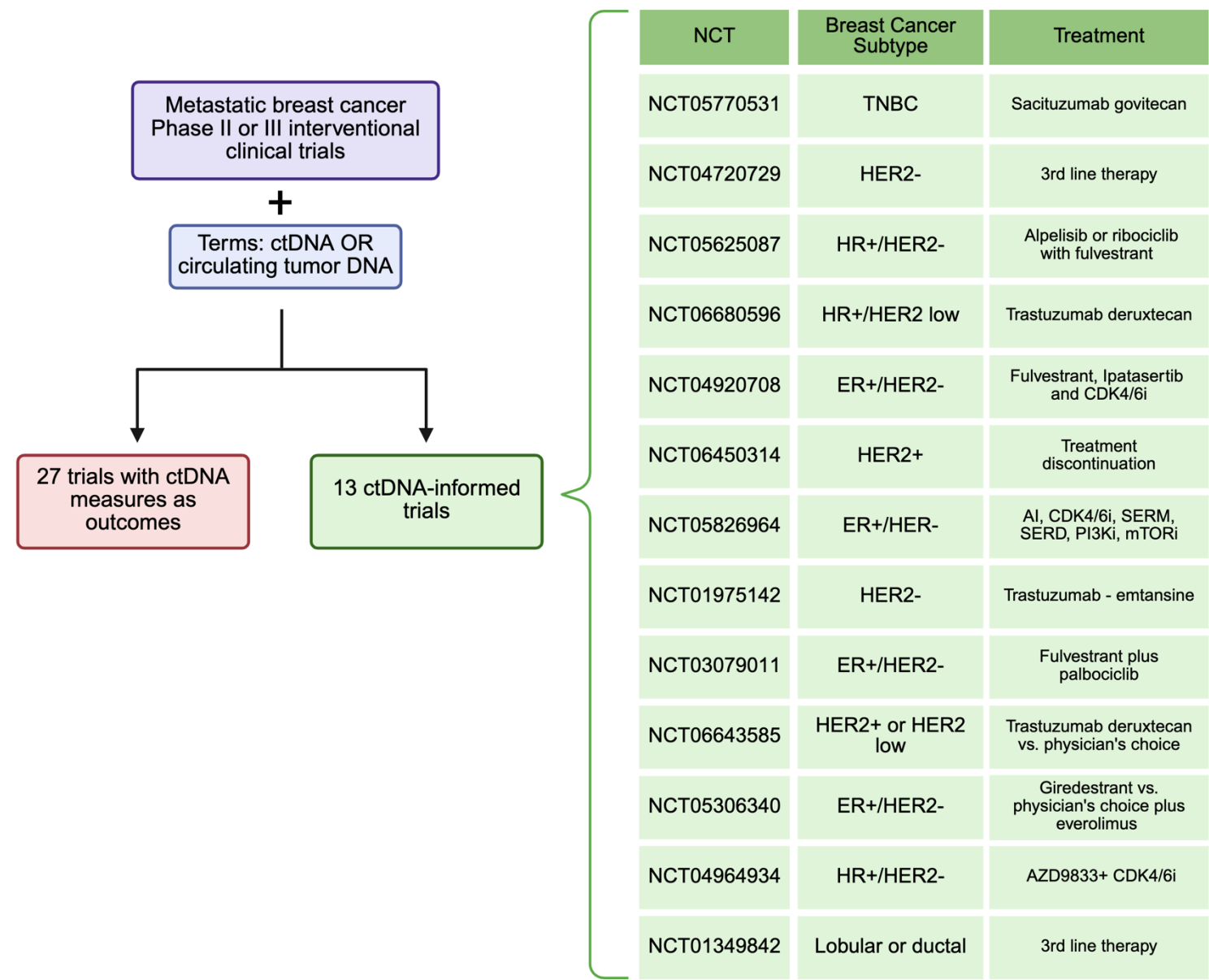

Supplement: Supplementary Figure 1 — Phase II/III Clinical trials Utilizing ctDNA. Forty-four Phase II or III interventional trials in metastatic breast cancer were identified. Of these trials, 32 were measuring ctDNA as one of their outcomes or monitoring ctDNA over time, and 12 trials proposed to utilize ctDNA measures up-front to inform the interventional component of the study. Trials with any phase 1 component, as well as terminated or withdrawn trials were not included. [file Image1.pdf]
