# Supplementary material for: Circulating DNA tumor fraction as a biomarker for advanced breast cancer
Source: Front Oncol. 2025 Nov 10;15:1655415. doi: 10.3389/fonc.2025.1655415 (PMC12640844; doi:10.3389/fonc.2025.1655415)
Supplement: Supplementary Table 1 — Commercial liquid biopsy assay comparison. Examples of commercial assays vary in the number of genes evaluated and other reported metrics (not exhaustive list). The assays listed all report Tumor Fraction. Each assay has variable published concordance and positive percent agreement (PPA) data. [file Table1.docx]

**Supplementary Table 1. Commercial liquid biopsy assay comparison.** Examples of commercial assays vary in the number of genes evaluated and other reported metrics (not exhaustive list). The assays listed all report Tumor Fraction. Each assay has variable published concordance and positive percent agreement (PPA) data.

**
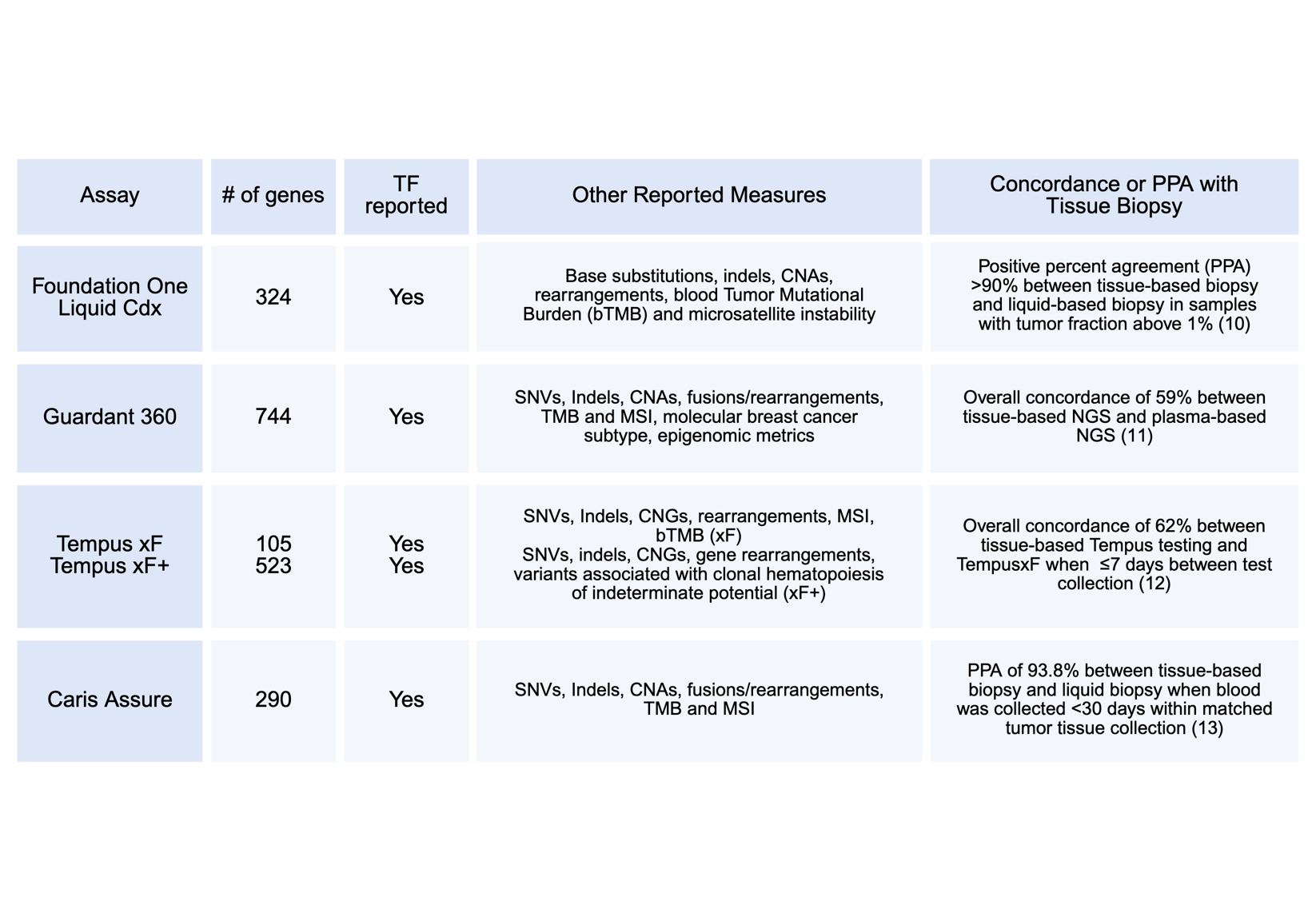
**
